# TP53 P72R Polymorphism Is Associated With P53 Protein Expression in Fatal Prostate Cancer: A Novel Case Report and Review of the Literature

**DOI:** 10.7759/cureus.94573

**Published:** 2025-10-14

**Authors:** Pooria Asili, Jordan Selep, Coen J Lap, Maneesh Jain, Victor E Nava

**Affiliations:** 1 Department of Pathology and Laboratory Medical Service, Veterans Affairs Medical Center, Washington, DC, USA; 2 Department of Hematology and Oncology, Veterans Affairs Medical Center, Washington, DC, USA; 3 Department of Hematology and Oncology, Veterans Health Administration, Washington, DC, USA; 4 Lombardi Comprehensive Cancer Center, MedStar Georgetown University Hospital, Washington, DC, USA; 5 Department of Pathology and Laboratory Medical Service, Veterans Health Administration, Washington, DC, USA; 6 Department of Pathology, George Washington University, Washington, DC, USA

**Keywords:** genetic alteration, immunohistochemistry, next-generation sequencing, p53, p72r polymorphism, prostate cancer

## Abstract

Somatic alterations of TP53 in prostate cancer (PCa) are linked to high-grade tumors, increased metastatic potential, and an adverse prognosis. Although germline TP53 mutations significantly increase the risk of developing aggressive PCa, the impact of single-nucleotide polymorphisms (SNPs) remains less understood. This report presents a novel case of a patient diagnosed post-mortem with aggressive, metastatic PCa, with autopsy revealing a TP53 variant at codon 72 as the sole abnormality with confirmatory histology, immunohistochemistry, and next-generation sequencing. The arginine variant (R72) is known to have altered biochemical properties and may increase the risk of aggressive PCa development. The impact of this polymorphism on PCa risk has not been fully established and remains controversial. This report reviews the existing literature on the TP53 P72R polymorphism and its association with PCa risk. Although the exact biological significance remains to be defined, the presence of this SNP might indicate an increased risk for the development of aggressive PCa in patients.

## Introduction

Prostate cancer (PCa) is the second most common cancer and fifth leading cause of cancer-related deaths among men globally, resulting in over 1.4 million diagnoses and nearly 400,000 deaths in 2022 [1]. The disease is clinically diverse, ranging from indolent, localized tumors in the majority of patients, to aggressive, metastatic disease in approximately 10-20% of patients [1-3]. This inherent variability complicates accurate risk stratification, creating a long-standing, but unmet clinical need for reliable prognostic markers.

The TP53 gene, encoding the p53 tumor suppressor protein, is essential for genomic stability and regulates biological processes such as cell cycle arrest, DNA repair, and apoptosis [4,5]. While historically considered a late-stage event in PCa, somatic TP53 alterations are now also frequently recognized in localized and castration-sensitive metastatic stages, with increasing prevalence up to 50% in advanced and castration-resistant disease [6-8]. The presence of these abnormalities is associated with aggressive tumor phenotypes, increased metastatic potential, and poor prognosis, underscoring its significance as a prognostic marker [9-11]. Pathogenic and likely pathogenic germline variants of TP53 have been described, including R158H, R282Q, R283C, and R337H, causing autosomal dominant cancer predisposition syndromes (Li-Fraumeni syndrome), and increasing the risk of aggressive PCa 9.1-fold (95% CI: 6.2-14; p < 0.001) relative to population controls [12].

In addition to these germline mutations, studies have linked single-nucleotide polymorphisms (SNPs) in TP53 and its negative regulators with PCa susceptibility and aggressiveness [13,14]. Notably, two non-synonymous SNPs in TP53, P72R (rs1042522) and P47S (rs1800371), as well as SNPs in regulators MDM2 (rs2279744), MDM4 (rs1380576), and HAUSP/USP7 (rs1529916), have been associated with PCa development and progression [13]. However, the impact of the TP53 P72R SNP in PCa pathogenesis remains unclear. This SNP, located at codon 72 of exon 4, results in a proline (CCC) to arginine (CGC) change and functionally modifies the p53 protein, which may influence cancer risk, progression, and response to treatment [15,16].

This report describes a patient found post-mortem to have high-grade metastatic PCa with tumor p53 overexpression by immunohistochemistry (IHC) and a TP53 codon 72 variant identified by next-generation sequencing (NGS).

## Case presentation

**Figure 1:**
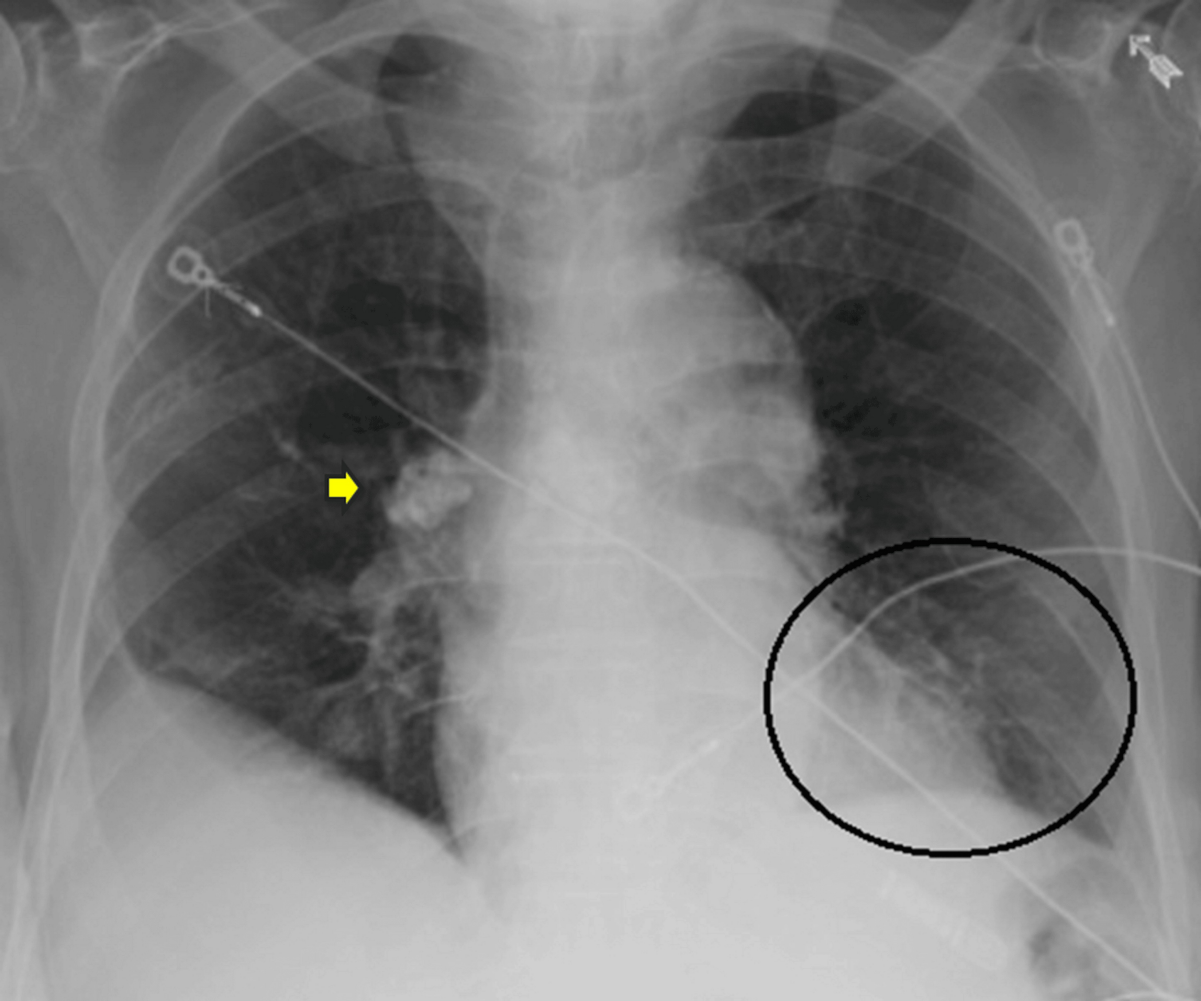
Chest X-ray demonstrating interval development of new non-specific left lower lung infiltrates (circled) and incidental right perihilar calcified lymph nodes (yellow arrow).

**Figure 2:**
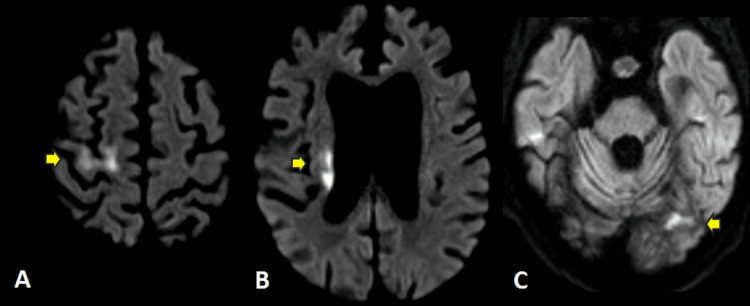
Brain MRI demonstrating restricted diffusion with diffusion-weighted imaging (DWI) hyperintense signal (yellow arrows) of the (A) right precentral gyrus, (B) right coronal radiata, and (C) left occipital lobe, concerning for acute infarctions.

An 87-year-old Caucasian male with a history significant for end-stage vascular dementia, ischemic strokes, atrial fibrillation, and benign prostate hyperplasia (BPH) presented in June of 2021 because of worsening encephalopathy. At presentation, a cachectic and frail, but afebrile patient was seen. Laboratory testing is summarized in Table 1. Normocytic anemia and mild thrombocytopenia were noted, with otherwise a normal white blood cell count. Other tests, including kidney, liver, and thyroid function, electrolytes, and acid-base status, were within normal limits. An EKG was consistent with atrial fibrillation. Chest X-ray showed new left-sided opacities and incidental right perihilar calcified lymph nodes (Figure 1). Brain MRI revealed acute infarcts of the right coronal radiata, right precentral gyrus, and left temporal occipital regions concerning for cardio-embolic etiology (Figure 2). He was admitted and started on broad-spectrum antibiotics for presumed left-sided pneumonia, with additional findings of acute cardio-embolic strokes. The encephalopathy was attributed to delirium secondary to an infection. During hospitalization, his clinical condition deteriorated, and after multi-disciplinary consultation, the decision was made to pursue comfort measures, and he eventually expired.

**Table 1:** Significant laboratory testing at the time of presentation.

Test (units)	Results	Reference range
Hemoglobin, g/dL	11.7	14.0-18.0
Mean corpuscular volume, fL	92.3	80-100
Platelet count, per cm³	6.7 x 10^3^	4-11 x 10^3^
White blood cell count, per cm³	114 x 10^3^	150-400 x 10^3^
Blood urea nitrogen, mg/dL	3	7-20
Creatinine, mg/dL	0.6	0.7-1.3
Estimated glomerular filtration rate, ml/min/1.73 m²	>60	>60
Sodium, mmol/L	134	135-145
Potassium, mmol/L	3.4	3.6-5.2
Bicarbonate, mmol/L	24.0	21-31
Chloride, mmol/L	103	100-109
Calcium, mg/dL	8.4	8.5-10.2
Albumin, g/dL	2.4	3.5-5.0
Glucose, mg/dL	90	70-121
Total bilirubin, mg/dL	0.4	0.2-1.2
Alanine aminotransferase, U/L	11	6-33
Aspartate aminotransferase, U/L	14	8-40
Thyroid-stimulating hormone, µIU/mL	2.3	0.45-4.5

**Figure 3:**
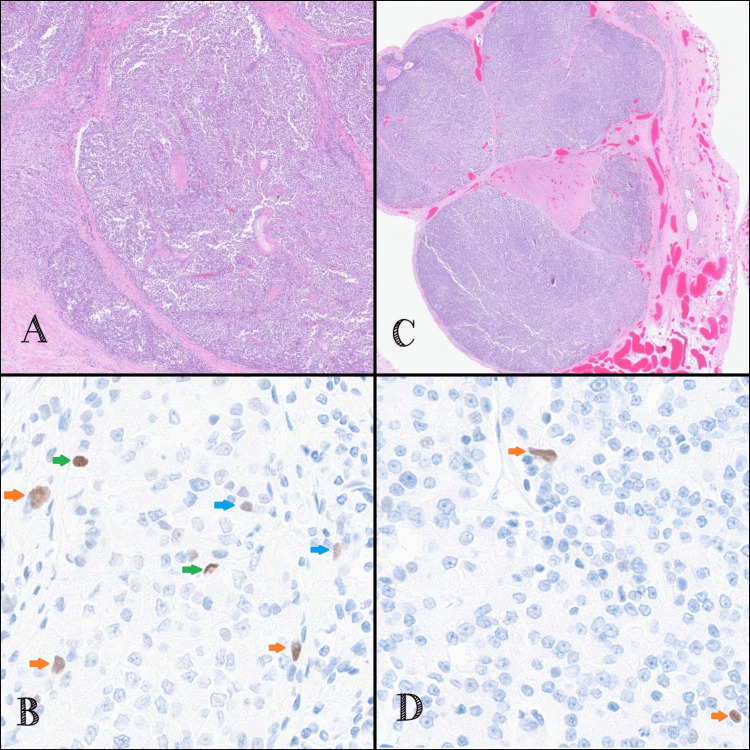
Hematoxylin & eosin (H&E) and p53 immunostaining of prostate primary and pleural metastasis. (A) Prostatic tumor. H&E staining (20x magnification). (B) P53 immunostaining of the prostate (400x magnification). (C) Pleural metastasis. H&E staining (20x magnification). (D) P53 immunostaining of pleural metastasis (400x magnification). (B, D) Blue, orange, and green arrows indicate low (1+), intermediate (2+), and strong (3+) P53 expression in a few tumor cells, respectively, based on brown nuclear staining with diaminobenzidine.

Autopsy was performed and revealed an enlarged prostate as well as multiple pulmonary and pleural nodules. Histologic sections of the prostate showed poorly differentiated adenocarcinoma composed predominantly of sheets of monotonous, medium-sized cells with ovoid nuclei, vesicular chromatin, occasional tiny nucleoli, and moderately abundant eosinophilic cytoplasm (Figure 3A). Areas of necrosis were noted. Well-controlled IHC revealed tumor cells positive for PSMA, PSAP, and racemase, and negative for PSA, P63, CK34BETAE12, and PAX-8. These pathological findings supported a diagnosis of prostatic acinar adenocarcinoma, Gleason score 4+5 (Grade Group 5). Histologic sections from pulmonary nodules, pleural nodules, and bone marrow revealed neoplastic cells with similar morphology and immune profile as the prostate tumor, consistent with metastatic disease (Figure 3C). Suppurative inflammation characteristic of bronchopneumonia was not observed, suggesting either interval resolution after antibiotic treatment or atelectasis seen on prior imaging. IHC performed on both the primary and pleural tumors revealed lympho-vascular invasion with podoplanin/D2-40. Although less than 1% of tumor cells stained positive for p53, there were some stained tumor cells with the intensity equivalent to strong (3+), intermediate (2+), and low (1+) in the primary tumor (Figure 3B), and intermediate (2+) in the pleural metastasis (Figure 3D). NGS performed on sections of a benign lymph node and the primary prostate tumor revealed a TP53 variant (c.215C>G; p.Pro72Arg; P72R; rs1042522), with a variant allele frequency of 47.06% in the benign tissue and 18.63% in the tumor. No other molecular abnormalities were identified, specifically no other alterations in TP53.

Retrospective chart review revealed that he was evaluated back in 2002 by a urologist for hematuria in the setting of kidney stones, and during that time was found to have BPH with a low prostate-specific antigen (PSA) level. His PSA had gradually trended up through 2007-2015, reaching 10.8 ng/mL in June of 2015 with increased PSA velocity (6.1 ng/mL/year). Although further evaluation was recommended, this was declined by the family in view of multiple comorbidities and advanced age. No PSA was obtained since 2015.

## Discussion

Our patient was found post-mortem to have metastatic PCa with neoplastic cells in the primary tumor and metastatic deposits expressing p53 protein. NGS on tumor tissue detected a variant in codon 72 of TP53, which was also identified in benign tissue, suggesting it to be a well-described SNP. Research has shown that detection of focal p53 nuclear expression by IHC in PCa is suggestive of the presence of pathogenic TP53 alterations and predicts aggressive disease [17-20]. Moreover, a recent study showed that strong (3+) p53 nuclear intensity of any percentage is significantly associated with Grade Group, biochemical recurrence, and development of metastatic progression in PCa [20]. Despite wide coverage of the TP53 gene, including all coding regions, no mutations in TP53 were detected, and the P72R was the only genetic abnormality identified. Although it remains speculation, our findings suggest that the P72R could underlie the observed p53 expression.

SNPs are common DNA variations that are defined as single base pair changes occurring in over 1% of the population [21]. Although over 200 SNPs have been identified in TP53 in population studies, the clinical consequences of the majority remain unknown [22]. The P72R is the most frequently reported SNP in TP53 with a frequency that varies among ethnicities. Whereas the arginine variant (CGC; R72) is more common in Caucasians, the ancestral proline variant (CCC; P72) is more frequently observed in people of African and Asian descent [23]. Studies have shown that the P72R polymorphism is located in the proline-rich domain of the p53 protein, and the R72 variant alters cellular processes such as cell-cycle arrest, apoptotic potential, and inflammation [4,5,24]. Although genome-wide association studies have not been able to identify either P72 or R72 as major risk variants for cancer, studies investigating this SNP have revealed frequent associations with malignancies, including breast cancer, gastric cancer, leukemia, and lung cancer [25-28]. In PCa, studies reviewing the impact of the P72R polymorphism have yielded conflicting results. A selection of previous studies is presented in Table 2 [29-37]. While a meta-analysis of 22 case-cohort studies in 2019 did not find an association between codon 72 polymorphisms and PCa risk (Arg vs. Pro; OR: 1.12, 95% CI: 0.90-1.75), a recent observational cohort study showed a highly significant association between the R72 variant (G allele) and PCa risk (OR: 7.937; 95% CI: 5.37-11.0, p<0.0001) in a Caucasian population, but no statistically significant enrichment of the R72 variant in patients with higher Gleason score disease (≥8) [29,30].

**Table 2:** Selected studies on P72R SNP and PCa association. SNP: single nucleotide polymorphism; PCa: prostate cancer; CI: confidence interval; OR: odds ratio; p: p-value; RR: relative risk.

Author/year	Study design	Sample size	Relevant findings
Toscano-Guerra et al. (2024) [29]	Observational cohort study	12 hormone-naive aggressive PCa cultures, 11 radical prostatectomies, and 94 serum samples	Significant association between the rs1042522 P72R SNP (G allele) and PCa risk with OR = 7.937 (95% CI: 5.37-11.0; p<0.0001) in a Caucasian population. Pronounced prevalence of the arginine allele in patients with higher Gleason scores (≥8).
Han et al. (2019) [30]	Meta-analysis of 22 case-control studies	3,146 PCa cases and 4,010 controls	No association was observed between the codon 72 polymorphism and PCa risk (Arg vs. Pro; OR = 1.12, 95% CI: 0.90-1.75), with consistent findings in subgroup analysis based on ethnicity.
Lu et al. (2014) [31]	Meta-analysis of 17 case-control studies	2,371 PCa cases and 2,854 controls	P72R polymorphism was not associated with PCa risk in all genetic models. When limited to studies confirming to Hardy-Weinberg equilibrium; a significant association was seen in the Caucasian population between Pro/Pro (C/C) vs. Arg/Arg (G/G) genotype (OR = 1.57, 95% CI: 1.08-2.28; p =0.017) and Pro/Pro (C/C) vs. Arg/Pro (G/C) + Arg/Arg (G/G) (OR = 1.60, 95% CI: 1.12-2.27; p = 0.009).
Khan et al. (2014) [32]	Case-control study	146 PCa cases and 107 controls	The arginine allele (G allele) was significantly associated with an increased risk of PCa (OR = 3.54, 95% CI: 2.13-5.89; p<0.001) in a Pakistani population.
Zhang et al. (2011) [33]	Meta-analysis of 10 case-control studies	1,196 PCa cases and 1,704 controls	No significant differences in PCa risk were found between codon 72 polymorphism (Pro/Pro vs. Arg/Arg, RR: 1.12, 95% CI: 0.74-1.70; Pro/Pro + Pro/Arg vs. Arg/Arg, RR: 1.05, 95% CI: 1.00-1.11). In the stratified analysis by ethnicity, the same results were found.
Ricks-Santi et al. (2010) [34]	Case-control study	266 PCa patients and 196 controls	C/G and G/G genotypes were significantly associated with increased PCa risk (OR = 1.53, 95% CI: 1.02-2.29, p=0.04) in an African American population. After age-adjustment, association with PCa risk remained, but was not significant (OR = 1.48, 95% CI: 0.95-2.231; p=0.08).
Zhang et al. (2010) [35]	Meta-analysis of 6 case-control studies	582 PCa cases and 1,075 controls	No overall association between codon 72 polymorphisms and PCa risk was observed for Arg/Arg (G/G) vs. Pro/Pro (C/C) (OR = 0.88; 95% CI: 0.62-1.25) and Arg/Arg (G/G) vs. Arg/Pro (G/C) + Pro/Pro (C/C) (OR = 1.05; 95% CI: 0.78-1.43). In the subgroup analysis, Caucasians with the arginine (G) variant might have increased susceptibility Arg/Arg (G/G) + Arg/Pro (G/C) vs. Pro/Pro (C/C) (OR = 3.43; 95% CI: 1.13-10.39).
Suzuki et al. (2003) [36]	Case-control study	114 PCa cases and 105 controls	Adjusted OR for PCa with the Arg/Arg (G/G), Arg/Pro (G/C), and Pro/Pro (C/C) were 1.0 (95% CI: 0.53-1.88), 0.99 (95% CI: 0.53-1.88), and 2.80 (95% CI: 1.04-7.53) in a Japanese population. A greater number of patients with low-grade cancer were seen with Pro/Pro (OR = 0.41, 95% CI: 0.13-1.30, p=0.13).
Henner et al. (2001) [37]	Case-control study	115 PCa cases and 181 controls	Pro/Pro (C/C) genotype was associated with a significantly lower risk of prostate cancer (OR = 0.23, 95% CI: 0.07-0.79, p=0.012).

Studies suggest that the preferential loss of the P72 variant and retention of the R72 variant of TP53 holds biological significance for cancer initiation and progression [38-40]. Importantly, loss of heterozygosity (LOH) has been observed in tumor tissue, suggesting that the R72 variant could be selected for during tumor development [29]. The low variant allele frequency of the R72 variant observed in tumor tissue in our case could be suggestive of LOH, although this cannot be definitively concluded. The intense nuclear uptake of p53 observed with IHC is suggestive of mutant activity [41]. As a result of differences in the biological properties of the R72 variant, the accumulation of p53 in the nucleus would not be an unexpected finding. Alternatively, retention of the R72 variant has been shown to be able to modify the tumor suppressor activities of mutant p53 in human cancer if present [42-44]. However, no other mutations in TP53 were identified in our case.

## Conclusions

In summary, this case highlights, to the best of our knowledge, the first instance of a patient diagnosed post-mortem with aggressive metastatic PCa, where autopsy revealed the TP53 P72R variant as the sole abnormality confirmed by NGS in conjunction with protein overexpression of tumoral p53 by IHC. Due to conflicting data regarding the impact of the P72R SNP and PCa, screening of the general population is not recommended. While there might be a role for this SNP in PCa, its utility for population screening would not be high enough to justify the cost and potential anxiety associated with it. However, the discussion around this SNP does highlight how genetic background could potentially modulate aggressiveness and prognosis of cancers. Although further research is needed, it is possible that the presence of a TP53 P72R SNP could inform personalized treatment decisions in patients with PCa in the future.
